# Surgical Antimicrobial Prophylaxis in Patients of Neonatal and Pediatric Age Undergoing Orthopedic and Hand Surgery: A RAND/UCLA Appropriateness Method Consensus Study

**DOI:** 10.3390/antibiotics11030289

**Published:** 2022-02-22

**Authors:** Francesca Opri, Sonia Bianchini, Laura Nicoletti, Sara Monaco, Roberta Opri, Marilia Di Pietro, Elena Carrara, Erika Rigotti, Cinzia Auriti, Caterina Caminiti, Daniele Donà, Laura Lancella, Andrea Lo Vecchio, Simone Pizzi, Nicola Principi, Alessandro Simonini, Simonetta Tesoro, Eisabetta Venturini, Alberto Villani, Annamaria Staiano, Leonardo Marchesini Reggiani, Susanna Esposito, on behalf of the Peri-Operative Prophylaxis in Neonatal and Paediatric Age (POP-NeoPed) Study Group

**Affiliations:** 1 Pediatric Unit, Department of Surgical Sciences, Dentistry, Gynecology and Pediatrics, University of Verona, 37124 Verona, Italy; opri.francesca@gmail.com (F.O.); roberta.opri@gmail.com (R.O.); marilia.dipietro@studenti.univr.it (M.D.P.); erika.rigotti@aovr.veneto.it (E.R.); 2 Pediatric Clinic, University Hospital, Department of Medicine and Surgery, University of Parma, 43126 Parma, Italy; bianchini.sonia@outlook.it (S.B.); laura.nicoletti@studenti.unipr.it (L.N.); s.monaco1410@gmail.com (S.M.); 3 Infectious Diseases Section, Department of Diagnostics and Public Health, University of Verona, 37134 Verona, Italy; elena.carrara@univr.it; 4 Neonatology and Neonatal Intensive Care Unit, IRCCS Bambino Gesù Children’s Hospital, 00165 Rome, Italy; cinzia.auriti@opbg.net; 5 Research and Innovation Unit, University Hospital of Parma, 43126 Parma, Italy; ccaminiti@ao.pr.it; 6 Division of Paediatric Infectious Diseases, Department for Woman and Child Health, University of Padua, 35100 Padua, Italy; daniele.dona@unipd.it; 7 Paediatric and Infectious Disease Unit, Academic Department of Pediatrics, IRCCS Bambino Gesù Children’s Hospital, 00165 Rome, Italy; laura.lancella@opbg.net (L.L.); alberto.villani@opbg.net (A.V.); 8 Department of Translational Medical Science, Section of Pediatrics, University of Naples “Federico II”, 80138 Naples, Italy; andrealovecchio@gmail.com (A.L.V.); staiano@unina.it (A.S.); 9 Pediatric Anesthesia and Intensive Care Unit, Salesi Children’s Hospital, 60123 Ancona, Italy; pizzis@me.com (S.P.); dr.simonini@gmail.com (A.S.); 10 Università degli Studi di Milano, 20122 Milan, Italy; nicola.principi@unimi.it; 11 Division of Anesthesia, Analgesia, and Intensive Care, Department of Surgical and Biomedical Sciences, University of Perugia, 06129 Perugia, Italy; simonettatesoro@gmail.com; 12 Pediatric Infectious Disease Unit, Meyer Children’s Hospital, 50139 Florence, Italy; elisabetta.venturini@meyer.it; 13 Unit of Pediatric Orthopaedics and Traumatology, IRCCS Istituto Ortopedico Rizzoli, 40136 Bologna, Italy; leonardo.marchesinireggiani@ior.it

**Keywords:** antibiotics, hand surgery, orthopedic surgery, pediatric infectious diseases, surgical antibiotic prophylaxis

## Abstract

Surgical site infections (SSIs) represent a potential complication in any type of surgery and can occur up to one year after the procedure in the case of implant placement. In the field of orthopedic and hand surgery, the rate of SSIs is a relevant issue, considering the need for the placement of synthesis devices and the type of some interventions (e.g., exposed fractures). This work aims to provide guidance on the management of peri-operative antibiotic prophylaxis for the pediatric and neonatal population undergoing orthopedic and hand surgery in order to standardize the management of patients and to reduce, on the one hand, the risk of SSI and, on the other, the development of antimicrobial resistance. The following scenarios were considered: (1) bloodless fracture reduction; (2) reduction of unexposed fracture and grade I and II exposed fracture; (3) reduction of grade III exposed fracture or traumatic amputation; (4) cruel fracture reduction with percutaneous synthesis; (5) non-traumatic amputation; (6) emergency intact skin trauma surgery and elective surgery without synthetic media placement; (7) elective orthopedic surgery with prosthetic and/or synthetic media placement and spinal surgery; (8) clean elective hand surgery with and without bone involvement, without use of synthetic means; (9) surgery of the hand on an elective basis with bone involvement and/or with use of synthetic means. This manuscript has been made possible by the multidisciplinary contribution of experts belonging to the most important Italian scientific societies and represents, in our opinion, the most complete and up-to-date collection of recommendations regarding the behavior to be adopted in the peri-operative setting in neonatal and pediatric orthopedic and hand surgery. The specific scenarios developed are aimed at guiding the healthcare professional in practice to ensure the better and standardized management of neonatal and pediatric patients, together with an easy consultation.

## 1. Introduction

Surgical site infections (SSIs) represent a potential complication in any type of surgery and can occur up to one year after the procedure in the case of implant placement. SSIs are associated with prolonged hospital stay and increased postoperative mortality rates, also presenting a significant economic impact on the healthcare system [1,2]. The incidence and severity of SSIs depend on several factors, including the type of surgery, duration of the procedure, preoperative preparation, and most importantly, the patient’s underlying medical condition [3]. Adequate preventive measures, including peri-operative administration of antibiotics, can effectively reduce SSI occurrence, both in adults and children [4,5].

Most of the knowledge about the risk factors and prevention of SSIs involves studies in the adult population, whereas currently available data on the pediatric and neonatal populations are limited, resulting in difficulties in the management of SSI prophylaxis. In particular, in the field of orthopedic surgery, the rate of SSIs is a relevant issue, considering the need for the placement of synthesis devices and the type of some interventions (e.g., exposed fractures). In the first case, in arthroplasty procedures, it is the formation of a bacterial biofilm on the surface of the implanted devices that represents a risk factor for the onset of SSI [3,6]. In the second case, the direct communication with the external environment in exposed fractures causes contamination and, therefore, an increased risk of subsequent wound infection. Moreover, the occurrence of an open fracture is not uncommon at a pediatric age, representing 0.7–2% of total fractures [3,7]. Hand surgery, even though it is included in orthopedic surgery, is often treated separately, considering the peculiarity of the anatomical district, the involvement of many structures, and the need to obtain optimal recovery of its function [6]. The microorganisms most frequently involved in SSIs following orthopedic and hand surgery are those of the skin flora, such as *Staphylococcus aureus*, Gram-negative bacilli, coagulase-negative *staphylococci* (including *S. epidermidis*), and β-hemolytic *Streptococci* [3].

This work aims to provide guidance on the management of peri-operative antibiotic prophylaxis for the pediatric and neonatal population undergoing orthopedic and hand surgery in order to standardize the management of patients and to reduce, on the one hand, the risk of SSI and, on the other, the development of antimicrobial resistance.

## 2. Methods

### 2.1. RAND/UCLA Method of Appropriateness

This paper was created using the RAND/UCLA (Research and Development Corporation and the University of California, Los Angeles) appropriateness method. This method consists of a panel of experts evaluating the appropriateness of diagnostic, management, and therapeutic procedures with suboptimal scientific evidence [8]. According to the RAND/UCLA appropriateness method, a procedure is defined as “appropriate” if the expected benefits outweigh the expected negative consequences. Conversely, a procedure whose expected risks outweigh the benefits is defined as inappropriate. According to the RAND definition, experts must make a judgment of appropriateness/inappropriateness by considering only the clinical benefits, disregarding any economic implications [9]. For a heterogeneous topic such as surgical antimicrobial prophylaxis (SAP), for which randomized controlled trials in pediatrics are lacking, the application of methods aiming to increase the homogeneity of behaviors by neonatologists, infectious disease specialists, pediatric surgeons, and anesthetists appeared useful and appropriate. For this reason, also considering recent positive experience in pediatrics [10,11,12], the RAND/UCLA approach was chosen instead of the GRADE methodology. Through the RAND method, the participants discussed different clinical scenarios and elaborated statements on the basis of the literature and their clinical experience.

### 2.2. Recruiting the Expert Panel

A multidisciplinary group of experts from the main Italian scientific societies was selected, composed of pediatricians, neonatologists, specialists in infectious diseases, pediatric surgeons, anesthesiologists, pharmacologists, and microbiologists. The following scientific societies were involved: Italian Society of Pediatrics (SIP), Italian Society of Neonatology (SIN), Italian Society of Pediatric Infectious Diseases (SITIP), Italian Society of Infectious and Tropical Diseases (SIMIT), Italian Society of Pediatric Surgery (SICP), Italian Society of Microbiology (SIM), Italian Society of Pharmacology (SIF), Italian Society of Neonatal and Pediatric Anesthesia and Resuscitation (SARNEPI), and Italian Society of Childhood Respiratory Diseases (SIMRI). The panel of experts was made up of 52 medical doctors with at least 5 years of experience: pediatricians (*n* = 20), neonatologists (*n* = 6), infectious disease specialists (*n* = 5), pediatric surgeons (*n* = 5), anesthetists (*n* = 8), pharmacologists (*n* = 5), and microbiologists (*n* = 3).

### 2.3. Scenario Formulation

A literature search was performed with a selection of papers, including randomized trials, systematic literature reviews, meta-analyses, and guidelines, on peri-operative prophylaxis to prevent SSIs during orthopedic surgery. The literature search was performed on the PubMed database, selecting English-language articles published from 2000 to 2021. Key search terms were: “antimicrobial prophylaxis” OR “antibiotic prophylaxis” AND “orthopedic” OR “fracture” OR “amputation” OR “prosthesis” OR “vertebral surgery” OR “hand surgery” AND “paediatric” OR “pediatric” OR “neonatal” OR “children”. Next, using the Patient/Problem/Population–Intervention–Comparison/Control/Comparator–Outcome (PICO) model, a questionnaire on peri-operative prophylaxis in orthopedic surgery was created for the pediatric and neonatal population, divided into nine clinical scenarios. Before administration, it was tested twice, with a one-week interval, among a convenience sample of 4 pediatricians, 2 neonatologists, one infectious disease specialist, one pediatric surgeon, one anesthetist, one pharmacologist, and one microbiologist. Then, 26 out of 52 experts were selected by the scientific societies for answering, and the questionnaire was administered to 11 pediatricians, 3 neonatologists, 2 infectious disease specialists, 3 pediatric surgeons, 4 anesthetists, 2 pharmacologists, and one microbiologist.

### 2.4. Two-Round Consensus Process

Based on the scenarios, a questionnaire was developed and submitted to experts on the online platform “REDCap”. Each question included the clinical scenario and possible answers were whether or not SAP was recommended for the scenario and, in case of its recommendation, a list with all the antibiotics available on the EU market so that the expert could select the antibiotics that he/she considered as the first choice. The selected literature material was made available to all panel members, who were instructed on how to complete the questionnaire. Experts responded to the questionnaire anonymously, and their judgment was expressed on a scale from 1 to 9, where “1” was considered definitely inappropriate, “5” uncertain, and “9” definitely appropriate. The intermediate values corresponded to different modulations of the judgment of inappropriateness (“2” and “3”), uncertainty (from “4” to “6”), and appropriateness (“7” and “8”). In evaluating each indication, each expert relied on both his or her own clinical judgment and experience and available scientific evidence. Free space was provided for any annotations or comments.

The first round of the questionnaire was conducted anonymously with respect to the other panel members. Multiple participation was not permitted by the platform, which guaranteed also the confidentiality and anonymity of the answers. Results of the survey were discussed in a collegial meeting with all 26 experts who answered the questionnaire to find an agreement and reduce eventual disagreement. Clarifications, adaptations, and refinements of the indications and appropriateness ratings were made. A total of 7 recommendations were developed (i.e., one for each scenario). All 52 participants were asked to approve the recommendations in a second round during the following four weeks.

## 3. Results

### 3.1. SCENARIO #1. Bloodless Fracture Reduction

Bloodless fracture reduction is a clean procedure, for which the most recent guidelines aimed at the adult population do not recommend peri-operative antibiotic prophylaxis [3]. There are no specific guidelines for neonates and children. The experts’ panel concludes that, for pediatric patients, the same recommendations considered for adults could be followed.

***Recommendation 1.*** Peri-operative antibiotic prophylaxis is not recommended in pediatric patients undergoing bloodless fracture reduction.

### 3.2. SCENARIO #2. Reduction of Unexposed Fracture and Grade I and II Exposed Fracture

Fractures are divided into unexposed and exposed. The former is presented with intact overlying skin, while exposed ones are accompanied by a break in the skin, with communication outside the bone stump or continuity with an associated hematoma. These are classified into grade I, II, and III based on contamination, soft tissue damage, and complexity. Referring to the Gustilo–Anderson classification, which is applicable to adult and pediatric populations, grade I and II fractures have skin lesions without significant surrounding soft tissue compromise [3]. There is strong evidence that timely antibiotic treatment plays a significant role in reducing the risk of deep infection [13]. Studies in the pediatric population showed that the risk of infection varies significantly based on fracture severity, being significantly higher in grade III exposed fractures than in grade I and II fractures [14,15]. In the adult population, many authors recommend, for less severe (grade I and II) exposed fractures, coverage only for Gram-positive microorganisms with the administration of a cephalosporin [16], while others opt for broader coverage of Gram-negative pathogens as well [17]. In an extensive review on the management of exposed fractures in the adult population, Garner and colleagues report that the Eastern Association for the Surgery of Trauma (EAST) recommends coverage of Gram-positive bacteria with systemic antibiotics as antibiotic prophylaxis in open fractures [18]. The use of first-generation cephalosporins was also advocated in 2011 by the Surgical Infection Society [19].

***Recommendation 2.*** In pediatric patients undergoing emergency surgery for the reduction of an unexposed fracture with an open approach and emergency surgery for the reduction of grade I and II exposed fractures, peri-operative antibiotic prophylaxis with cefazolin with a single dose of 30 mg/Kg (maximum dose 2 g) EV is recommended within 30 min before surgery and repeatable in case of surgery lasting more than 4 h.

### 3.3. SCENARIO #3. Reduction of Grade III Exposed Fracture or Traumatic Amputation

According to the Gustilo–Anderson classification, exposed fractures with extensive soft tissue damage, resulting in the risk of extensive contamination of the surgical site, are defined as grade III [3]. Typically, management involves empiric antibiotic prophylaxis associated with surgical debridement [3]. Most authors agree on the timely administration of antibiotic prophylaxis in the pediatric and adult population, whereas the ideal duration of postoperative administration remains controversial [7].

In the aforementioned review by Garner and colleagues, the authors reported that in grade III exposed fractures, prophylaxis against Gram-positive pathogens should be added to prophylaxis against Gram-negative pathogens [18]. They also reported the possibility of performing high-dose penicillin therapy for lesions potentially contaminated with soil or feces [18,20].

In a 2016 comparative study, no different rates of SSIs were shown in patients with exposed grade III fractures who had received antibiotic prophylaxis with a combination of cefazolin and gentamicin compared with piperacillin/tazobactam [21]. The use of fluoroquinolones also showed no advantage over cephalosporin/aminoglycoside regimens, in addition to the potential risk for fracture healing of these antimicrobials [16].

***Recommendation 3.*** In the pediatric patient undergoing emergency surgery for the reduction of an exposed grade III fracture or traumatic amputation, peri-operative prophylaxis with cefazolin at a dose of 30 mg/Kg (maximum dose 2 g) IV is recommended within 30 min before surgery, repeatable in case of surgery lasting more than 4 h, except in cases where broad-spectrum antibiotic therapy is already in progress.

### 3.4. SCENARIO #4. Cruel Fracture Reduction with Percutaneous Synthesis

In cruel fracture reduction procedures with percutaneous synthesis, the implants used and the peri-prosthetic microenvironment represent risk factors for SSIs [22]. The pathogen most commonly responsible for SSI in these procedures is *S. aureus* [23].

In an extensive review on the prevention of SSIs in orthopedic surgery, Tucci and colleagues state that antibiotic prophylaxis, with first/second-generation cephalosporins as the first choice, is recommended in orthopedic and traumatic surgery requiring device implantation by open surgery (joint replacement, osteosynthesis). A first antibiotic dose within 30–60 min of incision with first/second-generation cephalosporins is recommended, and possible additional dosing if surgery is prolonged [6]. In agreement, Bratzler and colleagues report cefazolin as the molecule of choice for prophylaxis in orthopedic procedures involving internal fixation surgery [3]. It is worth reporting that Xu and colleagues propose the application of a risk assessment in adults who are candidates for internal fracture reduction and stabilization [22]. This assessment, which takes into account patient health status, tissue and wound conditions, duration of surgery, and type of implanted devices, aims to identify low-risk patients for whom the incidence of SSI is low and, compared with untreated controls, not reduced by antibiotic prophylaxis with cefuroxime [22].

***Recommendation 4.*** In the pediatric patient undergoing emergency surgery for the cruel reduction of a fracture with percutaneous synthesis (e.g., Kirschner wires), peri-operative prophylaxis with cefazolin at the dose of 30 mg/Kg (maximum dose 2 g) IV is recommended within 30 min before surgery, repeatable in case of surgery lasting more than 4 h, and to be administered every 8 h for 24 h.

### 3.5. SCENARIO #5. Non-Traumatic Amputation

No guidelines are currently available for the management of peri-operative antibiotic prophylaxis in amputation procedures in the pediatric population. In the case of major amputations (e.g., limb), stump infection is common and can cause re-amputation, exposing the patient to further serious complications [24].

There are randomized trials in the adult population that evaluated the usefulness of antibiotic prophylaxis versus control. A significant reduction in stump infection rates and a reduced rate of re-amputation has been found in the case of antibiotic use, indicating the need for prophylactic antibiotic administration [25,26,27,28]. In addition, particular attention should be paid to methicillin-resistant *S. aureus* (MRSA) infections, which increase the risk of complications and postoperative death, suggesting the need to identify patients with MRSA colonization before surgery so that eradication could be attempted [29,30,31,32].

Although the indication of prophylactic use of antibiotics in this type of surgery is clearly defined, the type of antibiotic and duration of postoperative treatment remain widely debated. Some studies suggest that a 5-day course of antibiotics may increase the chance of primary healing and reduce hospital stays [33,34]. Banza and suggest that, in the case of amputation procedures in the pediatric population, antibiotic prophylaxis should always be performed to prevent SSIs, even when the procedure falls into the “clean” category [35]. The Colombian guidelines published in 2015 recommend antibiotic prophylaxis with cefazolin (four doses in total to be administered in 24 h) [36]. Moreover, in the case of amputation motivated by intractable limb infection, it is recommended to continue the antibiotic therapy already in place in association with cefazolin, with a single dose 30–60 min before the skin incision; subsequently, antibiotic therapy should be continued for 2–5 days in the postoperative phase [36]. Our experts’ panel agreed on the peri-operative prophylaxis with cefazolin IV without administration of postoperative antibiotics in the absence of infections.

***Recommendation 5.*** In the pediatric patient undergoing elective amputation surgery, peri-operative prophylaxis with cefazolin at a dose of 30 mg/Kg (maximum dose 2 g) IV is recommended to be administered within 30 min before surgery, repeatable if the surgery lasts longer than 4 h. In the case of intractable limb infection, the latter prophylaxis should be added to the antibiotic treatment already in place.

### 3.6. SCENARIO #6. Emergency Intact Skin Trauma Surgery and Elective Surgery without Synthetic Media Placement

Regarding clean orthopedic surgical procedures that do not involve the placement of synthetic materials, the most recent guidelines aimed at the adult population do not recommend peri-operative antibiotic prophylaxis [3,37]. No data are available for the pediatric population. The experts’ panel concluded that, for neonatal and pediatric patients, the same recommendations considered for adults could be followed.

***Recommendation 6***. No antibiotic prophylaxis is recommended in neonatal and pediatric patients undergoing emergency intact skin soft tissue trauma surgery and elective orthopedic surgery without placement of synthetic media.

### 3.7. SCENARIO #7. Elective Orthopedic Surgery with Prosthetic and/or Synthetic Media Placement and Spinal Surgery

When evaluating prophylaxis for procedures involving the implantation of fixation devices, the use of antibiotics should be considered because of the associated morbidity and costs of any infections to the implanted devices [38]. Although the CDC has defined deep SSIs as presenting within the first 90 days after surgery, infections can develop even later, with challenging consequences, including removal of implants, with subsequent accentuation of deformities [1].

Gatell and colleagues demonstrated a statistically significant decrease in the incidence of superficial wound infections in patients who had received an Ender’s or Kuntscher’s nail, bone plate, or other internal fixation device following preoperative cefazolin administration [39]. Current indications in the pediatric population are derived from recommendations provided by adult studies, indicating cefazolin as the antibiotic to be administered as prophylaxis [3]. The duration of surgery, particularly if longer than 2 h, has been identified in several papers as a risk factor for SSI. For this reason, some authors recommend the use of peri-operative prophylaxis in subjects who will undergo surgery of longer duration [40]. Kanj’s group, reviewing the literature, concluded on the non-necessity of using vancomycin to reduce the incidence of SSIs in clean orthopedic surgeries [41].

Looking specifically at spine surgery, the rate of postoperative SSI after scoliosis surgery ranges from 2.2 to 8.5%, including both superficial and deep infections [42]. In addition, Glotzbecker’s group reported the following risk factors for increased SSI in pediatric patients undergoing spinal surgery: urinary or bowel incontinence, positive urine culture preoperatively, inadequate antibiotic prophylaxis, use of prominent implants, and first-generation stainless steel implants (compared with titanium implants) [43]. Therefore, peri-operative prophylaxis with first-generation cephalosporin (e.g., cefazolin) at the dosage of 30 mg/Kg is suggested for this type of spine surgery [37,44,45].

***Recommendation 7.*** In neonatal and pediatric patients undergoing elective orthopedic surgery with prosthesis placement, orthopedic surgery with synthesis device placement, and elective orthopedic spine surgery with synthesis device placement, peri-operative antibiotic prophylaxis with cefazolin at a dose of 30 mg/Kg (maximum dose 2 g) IV is recommended within 30 min before surgery, repeatable in case of surgery lasting more than 4 h.

### 3.8. SCENARIO #8. Clean Elective Hand Surgeries with and without Bone Involvement, without Synthetic Means

After elective hand surgery procedures, SSIs are rare but can cause non-negligible morbidity [46]. Possible sequelae include increased fibrosis, stiffness, and contracture at the wound site, which can lead to significant hand malfunction, resulting in the need for rehabilitation and delayed return to work [47]. Bykowski’s group analyzed 8850 clean hand surgeries and reported an overall SSI rate of 0.35%, with no statistically significant differences between those who had received peri-operative antibiotic prophylaxis and those who had not [48].

The literature mainly reports indications related to traumatic hand surgery and carpal tunnel surgeries (one of the most common procedures performed in hand surgery), while there are less data related to antibiotic prophylaxis in different hand surgery procedures involving soft tissues. Looking at infections, all considered superficial, at 30 days after surgery, the authors reported no statistically significant differences between the group that had received peri-operative prophylaxis and the group that had not [49].

Carpal tunnel syndrome represents the most common compression neuropathy of the upper extremities; failure of conservative therapy is an indication for surgery. The Harness group used the CDC definition of SSIs (i.e., superficial incision infection, deep incision infection, surgical site infection involving organs) to evaluate the incidence of infection after carpal tunnel surgery, reporting no statistically significant differences between the use of peri-operative prophylaxis (performed in 47% of total cases, without distinction by type and dosage of drugs) and the non-use of this, even in diabetic subjects. Therefore, the authors conclude that there is no need for routine peri-operative antibiotic prophylaxis, which should be considered in individual subjects based on the individual risk/benefit ratio [46]. The CDC findings indicate no evidence either supporting or disfavoring the use of peri-operative antibiotic prophylaxis [1]. Even the NICE guidelines do not recommend peri-operative antibiotic prophylaxis in procedures considered clean [50,51]. Platt and Page analyzed results from a survey by the British Society for Surgical of the Hand to evaluate the use of peri-operative prophylaxis in different types of surgery, noting extreme variability and highlighting the need for guidelines for uniform behavior [38]. Bratzler and colleagues recommend against the use of peri-operative prophylaxis in clean hand surgeries in which foreign body insertion is not anticipated [3].

Ayden’s group organized a prospective, double-blind study involving 1340 young adults (mean age 30.92 ± 2.04 years) undergoing both elective and emergency hand surgery to evaluate the efficacy of peri-operative antibiotic prophylaxis (with cefazolin 2 g, within 5 min to 2 h before the start of surgery, with additional doses every 4 h until the first 24 h) versus placebo. The authors observed no statistically significant differences between the two study arms in terms of the incidence of infection, adjusting the data based on length and type (dirty vs. clean) of surgery, concluding on the non-indication of routine peri-operative antibiotic prophylaxis to be considered in at-risk subjects, such as immunocompromised ones [52].

The recent extensive meta-analysis by Li and colleagues concluded no difference in the incidence of SSIs between subjects who received antibiotic prophylaxis and those in whom it was not performed [53]. Formaini’s group also reached the same conclusion: no increase in the incidence of SSI in pediatric patients undergoing minimally invasive orthopedic procedures (arthroscopies, percutaneous fixation procedures, carpal tunnel, trigger finger, soft tissue surgeries) in whom peri-operative antibiotic prophylaxis was not performed [54].

Even in subjects who have previously undergone total joint arthroplasty, in the case of clean hand surgery, Warnick’s group does not indicate the need for peri-operative prophylaxis to reduce the risk of prosthesis infection [55].

In an extensive meta-analysis, Murphy’s group also concluded that peri-operative antibiotic prophylaxis was not useful in reducing SSI incidence in superficial hand wounds requiring intervention [56]. A similar conclusion was reached by the Consensus of the American Association of Plastic Surgeons [57].

***Recommendation 8.*** No antibiotic prophylaxis is recommended in neonatal and pediatric patients undergoing elective clean soft-tissue hand surgery in the absence of bone involvement and elective hand surgery with bone involvement without the use of synthetic means.

### 3.9. SCENARIO #9. Surgery of the Hand on an Elective Basis with Bone Involvement and/or with the Use of Synthetic Means

Table 1 summarizes the main types of hand surgery, divided on the basis of the use or not of synthetic means.

As previously reported, the routine use of peri-operative antibiotic prophylaxis is not necessary for clean hand surgery without implants. In contrast, in surgeries in which metal implants are to be placed, several authors agree on the use of peri-operative prophylaxis. Dunn’s group indicates the need for prophylaxis for surgeries in which external fixators are used and/or Kirschner wires are applied [58]. In support of the utility and safety of peri-operative prophylaxis in these types of procedures, Sandrowski’s group reported a low incidence of antibiotic-prophylaxis-related adverse events (1.5%), predominantly cefazolin (and clindamycin, in cases with a positive history of penicillin and cephalosporin allergy) and mild [59]. However, peri-operative prophylaxis should not be prolonged, given the lack of benefit, unless special cases are involved (immunocompromised patients, prolonged surgery, trauma present for more than 48 h) [60].

Takesue’s guidelines state that in cases of hand wounds, classified as contaminated or soiled, requiring emergency surgery, therapeutic (not prophylactic) dose antibiotics are generally administered peri-operatively [61].

***Recommendation 9.*** In pediatric patients undergoing elective hand surgery with bone involvement using synthetic means and clean elective hand surgery lasting more than 4 h, peri-operative antibiotic prophylaxis with cefazolin at a dose of 30 mg/Kg (maximum dose 2 g) IV is recommended within 30 min before surgery, repeatable in case of surgery lasting more than 4 h, and to be administered every 8 h for 24 h.

## 4. Discussion

The application of adequate peri-operative antibiotic prophylaxis in the field of orthopedic and hand surgery in neonatal and pediatric age represents a complex field of application and poor shared indications. For these reasons, we found it necessary to work toward the creation of shared expert recommendations.

In the context of the exposed fractures, the scenarios divide the fractures of I and II degree, from the fractures of III, more serious and with extensive involvement of soft tissues. Furthermore, the exposed fractures represent a particular case in the surgical field, in which the wound is often contaminated for the same dynamics of the trauma. For this reason, the literature focuses mainly on antibiotics that start at the patient’s admission and, therefore, are often already in place at the time of surgery. The panel of experts concluded for peri-operative antibiotic prophylaxis with cefazolin, in substantial agreement with the literature, except in cases in which, for the reasons stated above, there is not already an antibiotic therapy in place. Within the treatment of fractures, we also wanted to add, for greater completeness of the document, a specific recommendation regarding the bloodless reduction of fractures, which, by definition, does not involve surgery.

In the case of patients undergoing emergency cruciate fracture reduction with percutaneous synthesis, the expert panel concluded on the administration of peri-operative prophylaxis with first-generation cephalosporin (cefazolin). This is in line with what is recommended in the literature, regardless of the individual risk assessment of developing SSI, and is in disagreement with Tucci and colleagues, who proposed an individual risk assessment of developing SSIs, according to which peri-operative prophylaxis would not be necessary for patients with low risk [3,6].

Concerning amputations, particularly major ones, the experts’ panel recommended performing broad-spectrum prophylaxis with a first-generation cephalosporin. Existing studies regarding this type of surgery are somewhat limited and dated, all agreeing on the need for antibiotic prophylaxis in patients undergoing major limb amputation to reduce the risk of stump infections, re-amputation, and prolonged hospital stays [24]. However, the optimal duration of peri-operative prophylaxis/antibiotic therapy remains controversial. The indication expressed by the panel to use cefazolin in the peri-operative prophylaxis of patients undergoing orthopedic surgery with prosthesis implantation appears to be in line with data in the literature [3,62].

The expert panel concluded that there is no indication for routine peri-operative antibiotic prophylaxis in hand surgeries with soft tissue involvement, without prosthetic or synthetic insertion, in agreement with reports in the literature in studies that have demonstrated an absence of reduction in SSIs in antibiotic prophylaxed subjects undergoing various surgeries, such as carpal tunnel surgery, trigger finger, cyst excision, De Quervain’s tenosynovitis, and mass excision, surgeries considered to be clean and of short duration [38,40,46,48,49,63,64]. These recommendations are of fundamental importance for the correct behavior to be undertaken, especially in elective clean procedures on the hand, in which, although there is no indication for prophylaxis, this is still used in percentages varying between 10 and 30%, not without complications [65,66].

With regard to the indication of peri-operative prophylaxis in hand surgeries involving the insertion of synthetic put-downs, the panel came out in favor of peri-operative prophylaxis, in agreement with the present literature, specifying the use of cefazolin, thus unifying the indication that until now was variable [67,68].

Some experts carry out peri-operative antibiotic prophylaxis even in elective surgery with joint involvement without inserting devices in arthroscopies due to the high risk of bleeding [3]. Similarly, in open fractures, they differentiate those that arrive within 6 h of the trauma from those that arrive over 6 h after the trauma: the latter, in addition to standard prophylaxis depending on the degree of the fracture, then continue the therapy for a total of 3 days [3]. As regards bone grafts from a homologous bank, specific preparation is usually carried out and usually the check that is done in the room before implanting the graft is negative [3]. Therefore, no specific prophylaxis is normally done. Further studies on these topics are certainly needed. What should be emphasized is that, in orthopedic surgery, the long duration of the operation (>4 h) and the risk of abundant blood loss (>30% of the volume) often make it necessary to repeat a further dose of antibiotics [3].

Cefazolin is recommended for use at 25 mg/kg every 12 h in neonates with body weight under 2 kg at birth, whereas 25 mg/kg is recommended every 8 h for neonates of 8–28 days with body weight over 2 kg at birth [69]. Although the neonatal pharmacokinetics differs depending on gestational age, body weight, and days after birth, the panel of experts did not recommend to change the cefazolin dose because of the short exposure duration of antimicrobial prophylaxis and the safety of cefazolin at neonatal age. On the other hand, the large majority of orthopedic and hand surgical procedures are performed in patients with a body weight ≥2 kg and beyond the neonatal age.

In the drafting of this work, the specific scenarios developed aimed at guiding the healthcare professional in practice to ensure the better and standardized management of neonatal and pediatric patients, together with an easy consultation. The strengths of the work are an updated review of the literature, the use of a rigorous method of analysis (RAND/UCLA), and the involvement of a large number of representatives of the most important Italian scientific societies. The potential limitation of the work is the scarcity of data in the literature and the main reference to adulthood, partly overcome by the involvement of many selected experts. Other limitations of the study included that this was an opinion-based survey to base recommendations and the agreement was reached in a collegial meeting. On the other hand, the lack of pediatric studies did not permit us to use the GRADE methodology and the complexity of the topic required an on-line, face-to-face meeting with all the participants. However, the RAND method did not permit us to define a hierarchy of antibiotics’ administration and not using the GRADE method may affect the quality of these recommendations. The findings obtained can establish the basis for educational interventions that aim to optimize the use of antibiotics in pediatric patients undergoing orthopedic and hand surgery.

## 5. Conclusions

This work has been made possible by the multidisciplinary contribution of experts belonging to the most important Italian scientific societies and represents, in our opinion, the most complete and up-to-date collection of recommendations regarding the behavior to be adopted in the peri-operative setting in neonatal and pediatric orthopedic and hand surgery. Table 2 summarizes the nine recommendations.

The application of uniform and shared protocols aims to improve the management of pediatric and neonatal patients, with, on the one hand, the possibility of reducing SSIs and, on the other hand, containing the phenomenon of antimicrobial resistance, with the consequent rationalization of resources and costs. Our panel of experts thinks that, in the face of extremely heterogeneous prescriptions in real life, characterized by the excessive and often inappropriate use of antibiotics in SAP, our document represents a balanced and shared text, derived from an extensive discussion, which can be extraordinarily beneficial for the patient and, more generally, for the health system. As soon as our Consensus document is implemented by the Italian Scientific Societies, it will be interesting to analyze its clinical and economic impact in our geographical context. However, our recommendations could be generalized also to low- and middle-income countries, where the impacts of simple, cost-effective, sustainable, and adaptable strategies on the reduction in morbidity risk and the associated costs have been recently highlighted [70].

## Figures and Tables

**Table 1:** Main hand surgeries, divided on the basis of the use or not of synthetic means.

Hand Operations without Insertion of Synthesis Media	Hand Operations with Insertion of Synthesis Media
- Carpal tunnel syndrome release surgery - Snap finger surgery - Hand surgery for dog and human bites - Excision of ganglion cysts - Release surgery in De Quervain’s syndrome - Release surgery in cubital tunnel syndrome - Dorsal and flexor tenosynovectomy of - wrist, palm and finger - Ulnar nerve transposition - Fasciotomy in Dupuytren’s contracture - Tendon Repair	- Proximal interphalangeal joint arthroplasty - Metacarpophalangeal joint arthroplasty - Thumb basal joint arthroplasty - Open reduction and internal fixation of distal forearm fracture - Arthroplasty of the carpo-metacarpal joint of the thumb.

**Table 2:** Surgical antimicrobial prophylaxis for patients of neonatal and pediatric age undergoing orthopedic and hand surgery.

Clinical Scenario	Recommendation
Bloodless fracture reduction	Peri-operative antibiotic prophylaxis is not recommended in pediatric patients undergoing bloodless fracture reduction.
Reduction of unexposed fracture and grade I and II exposed fracture	In the pediatric patient undergoing emergency surgery for reduction of an unexposed fracture with an open approach and emergency surgery for reduction of grade I and II exposed fracture, peri-operative antibiotic prophylaxis with cefazolin with a single dose of 30 mg/Kg (maximum dose 2 g) EV is recommended within 30 min before surgery and repeatable in case of surgery lasting more than 4 h.
Reduction of grade III exposed fracture or traumatic amputation	In the pediatric patient undergoing emergency surgery for reduction of an exposed grade III fracture or traumatic amputation, peri-operative prophylaxis with cefazolin at a dose of 30 mg/Kg (maximum dose 2 g) IV is recommended within 30 min before surgery, repeatable in case of surgery lasting more than 4 h, except in cases where broad-spectrum antibiotic therapy is already in progress.
Non-traumatic amputation	In the pediatric patient undergoing elective amputation surgery, peri-operative prophylaxis with cefazolin at a dose of 30 mg/Kg (maximum dose 2 g) IV is recommended to be administered within 30 min before surgery, repeatable if the surgery lasts longer than 4 h. In case of intractable limb infection, the latter prophylaxis should be added to the antibiotic treatment already in place.
Emergency intact skin trauma surgery and elective surgery without synthetic media placement	No antibiotic prophylaxis is recommended in neonatal and pediatric patients undergoing emergency intact skin soft tissue trauma surgery and elective orthopedic surgery without placement of synthetic media.
Elective orthopedic surgery with prosthetic and/or synthetic media placement and spinal surgery	In neonatal and pediatric patients undergoing elective orthopedic surgery with prosthesis placement, orthopedic surgery with synthesis device placement, and elective orthopedic spine surgery with synthesis device placement, peri-operative antibiotic prophylaxis with cefazolin at a dose of 30 mg/Kg (maximum dose 2 g) IV is recommended within 30 min before surgery, repeatable in case of surgery lasting more than 4 h.
Clean elective hand surgeries with and without bone involvement, without synthetic means	No antibiotic prophylaxis is recommended in neonatal and pediatric patients undergoing elective clean soft-tissue hand surgery in the absence of bone involvement and elective hand surgery with bone involvement without the use of synthetic means.
Surgery of the hand on an elective basis with bone involvement and/or with the use of synthetic means.	In pediatric patients undergoing elective hand surgery with bone involvement using synthetic means and clean elective hand surgery lasting more than 4 h, peri-operative antibiotic prophylaxis with cefazolin at a dose of 30 mg/Kg (maximum dose 2 g) IV is recommended within 30 min before surgery, repeatable in case of surgery lasting more than 4 h, and to be administered every 8 h for 24 h.

## Data Availability

All the data are included in the manuscript.
